# The Role of Brain-Derived Neurotrophic Factor in Comorbid Depression: Possible Linkage with Steroid Hormones, Cytokines, and Nutrition

**DOI:** 10.3389/fpsyt.2014.00136

**Published:** 2014-09-26

**Authors:** Tadahiro Numakawa, Misty Richards, Shingo Nakajima, Naoki Adachi, Miyako Furuta, Haruki Odaka, Hiroshi Kunugi

**Affiliations:** ^1^Department of Mental Disorder Research, National Center of Neurology and Psychiatry, National Institute of Neuroscience, Tokyo, Japan; ^2^Semel Institute for Neuroscience and Human Behavior, University of California, Los Angeles, Los Angeles, CA, USA; ^3^Department of Physiology, St. Marianna University School of Medicine, Kanagawa, Japan

**Keywords:** depression, BDNF, glucocorticoid, estrogen, cytokines, obesity, flavonoids

## Abstract

Increasing evidence demonstrates a connection between growth factor function (including brain-derived neurotrophic factor, BDNF), glucocorticoid levels (one of the steroid hormones), and the pathophysiology of depressive disorders. Because both BDNF and glucocorticoids regulate synaptic function in the central nervous system, their functional interaction is of major concern. Interestingly, alterations in levels of estrogen, another steroid hormone, may play a role in depressive-like behavior in postpartum females with fluctuations of BDNF-related molecules in the brain. BDNF and cytokines, which are protein regulators of inflammation, stimulate multiple intracellular signaling cascades involved in neuropsychiatric illness. Pro-inflammatory cytokines may increase vulnerability to depressive symptoms, such as the increased risk observed in patients with cancer and/or autoimmune diseases. In this review, we discuss the possible relationship between inflammation and depression, in addition to the cross-talk among cytokines, BDNF, and steroids. Further, since nutritional status has been shown to affect critical pathways involved in depression through both BDNF function and the monoamine system, we also review current evidence surrounding diet and supplementation (e.g., flavonoids) on BDNF-mediated brain functions.

## Introduction

In addition to genetic pre-dispositions, various environmental factors are also well-established to contribute to the pathogenesis of depressive disorders. Specifically, prolonged exposures to stressful environments, which result in chronic elevation of glucocorticoids, have been associated with depression onset (1). Generally, it is believed that glucocorticoid levels play several roles in coping with stressful conditions. It is well-established that, when receiving stressful stimuli, corticotropin-releasing hormone (CRH) is released in the hypothalamus, which stimulates the anterior pituitary to increase adrenocorticotropic hormone (ACTH) levels. Subsequently, ACTH acts on the adrenal glands to upregulate the concentration of glucocorticoids for coping with stress. However, one of the main causes of depression is hyperactivation of the hypothalamic–pituitary–adrenal axis (HPA axis) with persistently increased levels of glucocorticoids. Ample evidence demonstrates that glucocorticoid levels regulate neuronal transmission and synaptic plasticity (2), though abnormally elevated levels are recognized as a risk for depressive disorders (3). Thus, chronic glucocorticoid exposure has been utilized as a reliable animal model of depression (4). Estrogen, another steroid, also plays a role in neuronal plasticity, with altered levels proven to affect mood. Remarkably, adolescent offspring from mothers suffering from postpartum depression (PPD) exhibit higher susceptibility to depression compared to those from healthy mothers (5). Lower serum levels of estradiol were observed in women with major depressive disorder (MDD) (6), and low-plasma concentrations of estrogen were correlated with PPD (7). Although estrogen has been considered as a major factor in the pathophysiology of PPD, Wong et al. found that glucocorticoid levels also influence behaviors of mother rats and their subsequent pregnancy outcomes including litter size and the proportion of male/female sex (8), suggesting complex interactions among steroids in PPD. Furthermore, brain-derived neurotrophic factor (BDNF) plays an essential role in neuronal plasticity, with the downregulation of BDNF expression/function reproduced in a variety of animal models of depression. Thus, in this review, we provide current knowledge concerning dynamics of regulators for neuronal function (glucocorticoid, estrogen, BDNF, etc.) in PPD.

When considering comorbid depression, the possible relationship with pro-inflammatory cytokines is an important issue (9). Meta-analytic studies have demonstrated the association of pro-inflammatory cytokines such as tumor necrosis factor-α (TNF-α) and interleukin-6 (IL-6) with MDD (10, 11). In rats, acute intracerebroventricularly administration of IL-6 (50–200 ng) elicits depression-like behavior (12). It has been suggested that patients suffering from cancers (in which the inflammatory system is activated) have a higher prevalence of depressive symptoms (13). Consistently, interferon-α (INF-α), which is used as a cancer treatment, leads to depressive symptoms, suggesting involvement of cytokines in comorbid depression (14). Interestingly, nutritional and metabolic disease such as diabetes and obesity may also predispose individuals to depression (15). Oxidative insults, dysfunction of the HPA axis, inflammation, and reduced BDNF-related signaling have been identified as contributors to depression in obesity (15, 16). Remarkably, biological functions, including antioxidant (17), anti-cancer (18), and antidepressant-like effects (19) of flavonoids (major component of polyphenols extracted from plants) have been demonstrated. In the latter part of this paper, we discuss the relationship between inflammation, obesity, and overall brain function, introducing flavonoids as a potential drug for improvement of important neural processes.

## Basic Roles of BDNF in Neuronal Function

The neurotrophin family consists of nerve growth factor (NGF), BDNF, NT-3, and NT-4/5, which play a pivotal role in cell survival and synaptic function in the peripheral and central nervous systems. Members of the neurotrophin family bind with high affinity to their respective receptors, with NGF to TrkA, both BDNF and NT-4/5 to TrkB and NT-3 to TrkC (1, 20). Research has explored the interaction of neurotrophins with specific Trk receptors and their stimulation of downstream signaling pathways including phospholipase Cγ (PLCγ), phosphoinositide 3 kinase (PI3K)/Akt and the extracellular signal-regulated kinase (ERK) pathways that contribute to maintaining cell viability and regulation of synaptic function, although common low-affinity receptor p75 has a negative role in cell survival (1, 20). Among the entire neurotrophin family, BDNF/TrkB has been extensively examined regarding its positive effects including neuroprotection against cell death induction and on the development of neural circuits (21, 22). The Val66Met variation of BDNF is related with altered secretion of BDNF protein that influences structural alterations of brain tissue (23). In addition, it has been reported that genetic variant of TrkB is also associated with the risk of depression (24–26), further suggesting that the importance of BDNF/TrkB system in brain function.

## Influence of Estrogen on Neuronal Function

In addition to BDNF, the contribution of estrogen to neural function is well-established. The major forms of estrogen consist of estrone (E1), estradiol (E2, or 17β estradiol), and estriol (E3), which are mainly produced in the ovaries, corpus luteum, and placenta, through non-gonad organs such as liver and brain function are slight producers (27). Hippocampal long-term potentiation (LTP) is believed to be the basis of learning and memory. The form of estrogen is important for learning and memory, given that various doses of estrogen (E1, 17α estradiol, and E2) provide enhancement or impairment in the contextual fear conditioning in ovariectomized rats (28). Using acute hippocampal slices from ovariectomized rats, significant enhancement of LTP by 17β estradiol was revealed (29). In this model, alteration of spine density and *N*-methyl-d-aspartate (NMDA) glutamate receptor function were involved (29). Furthermore, activation of RhoA (small GTPase) and cofilin, as well as polymerization of actin, are putatively involved in enhanced neurotransmission caused by acute 17β estradiol application (30). In the learned helplessness model using ovariectomized rats, loss of LTP and decreased spine density in the hippocampus were observed in helpless animals compared with those of resilient animals (31). Importantly, replacement of 17β estradiol increased hippocampal LTP and spine density in both helpless and resilient rats (31).

Rodent studies have established a close relationship between estrogen and BDNF in the regulation of neuronal function. It has been demonstrated that 17β estradiol regulates mossy fiber neurotransmission in the hippocampus, and that not only mature BDNF/TrkB but also the precursor proBDNF/p75 system contributes to estradiol-mediated hippocampal function (32). Interestingly, in addition to classical nuclear receptors (ERα and ERβ), the extranuclear receptor for estrogen also plays a role in estrogen function. Using ovariectomized female mice, Yang et al. showed that E2 conjugates (binds to only extranuclear ER) protected neurons against global cerebral ischemia, and significantly increased phosphorylation of cAMP response element-binding protein (CREB) and BDNF expression via activation of ERK- and Akt-pathways (33). Currently, ample evidence suggests that 17β estradiol acutely affects synaptic plasticity via stimulating intracellular signaling such as the ERK pathway in a similar fashion as BDNF (34).

## Estrogen and Postpartum Depression

Depression is a critical problem for postpartum women, because PPD is thought to negatively influence both infant and mother. Indeed, adolescent offspring of PPD mothers have approximately five times greater risk of depression than offspring of healthy mothers (5). It is known that symptoms of PPD appear in about 10% of postpartum mothers and that PPD increases rates of suicide and consequent deaths (35). In addition, because infant care by the mother during the perinatal period influences mental health of offspring in adulthood (36), the possible mechanism underlying the onset of PPD should be elucidated.

Generally, it has been recognized that a greater frequency of women compared to men suffer from depressive disorders, with the postpartum phase being a high-risk period. As expected, alterations in estrogen-related genes and signaling are suspected to be involved in PPD. A recent study found an association between gene polymorphisms of estrogen receptor alpha and women with PPD (37). With a genome-wide association study, it was confirmed that sensitivity to estrogen-related signaling is increased in women with PDD (38). However, the association between estrogen levels and symptoms of PPD is still controversial. Mehta et al. reported that no alteration in plasma estrogen concentration is observed (38), whereas cross-species studies of PPD using rats and humans show a possible contribution of reduced estrogen to the pathogenesis of PPD (7). In addition to increased immobility of estrogen-withdrawal rats in the forced swim test (FST), a correlation between negative affect in 1-month postpartum women with PPD episode and their daily estradiol concentrations has been reported (7). Progesterone is highly produced sex hormone in the luteal phase, and its withdrawal is also suggested to be involved in PPD symptoms. Using the postpartum mouse model utilizing progesterone withdrawal, it is revealed that acute combined application of magnesium, zinc, and thiamine improve the depressive- and anxiety-like behaviors (39). Furthermore, blockade of progesterone receptor activity by the specific antagonist, CDB-4124, results in increased depressive-like behavior of female mice (40), supporting the hypothesis of progesterone withdrawal in PPD pathogenesis. Although detailed mechanism in the effect of some nutrients on progesterone ability is still unknown, it is interesting whether dietary supplementation attenuates the symptoms of PPD through the regulation of sex hormones such as progesterone.

Very recently, it has been reported that postpartum rats after chronic stress during their pregnant period exhibit depressive-like behavior, impaired ability to care for their young, and decreased spine density of pyramidal neurons in the medial prefrontal cortex (41). Wong et al. examined the possible impact of corticosterone administration during the first postpartum period on mother rats (8). Interestingly, increased depressive-like behavior of dams in the first postpartum period was observed along with a smaller litter size and less depressive-like behavior in the second postpartum period (8). As stressful conditions during pregnancy enhance the risk of depression, reduce maternal care, and negatively affect offspring characteristics, detailed molecular mechanisms behind PPD are desirable. Interestingly, Maniam and Morris showed that postpartum mother rats after daily long-term separation from pups display depressive-like and anxiety-like behaviors with increased plasma corticosterone, with high-fat diets (HFDs) improving these behaviors and steroid levels (42). A neurochemical approach using rats revealed that maternal isolation induces reduced activity of Na^+^, K^+^-ATPase in the hippocampus of postpartum mothers (43). Because the negative impact of maternal isolation on postpartum mothers is not well understood compared to offspring, increased PPD after maternal isolation is also remarkable. Importantly, it has been suggested that changes in the activity of Na^+^, K^+^-ATPase are involved in psychiatric diseases including bipolar disorder (44, 45). Focusing on the specific molecular pathways related to each disease is very important in future research. Although the influence of antidepressant treatment during pregnancy is a critical concern, there are few animal models for examination. Utilizing a rat model, Bourke et al. examined the effect of exposure to escitalopram [a selective serotonin reuptake inhibitor (SSRI)] during pregnancy, and found no substantial change in maternal care. To prevent negative effects on the mental and/or physical growth of offspring, further studies investigating prenatal antidepressant treatment are important (46). SSRIs are proven to be an effective treatment for PPD, though there is no sufficient evidence to present clear superiority of SSRIs (47). A recent randomized placebo-controlled, double-blind clinical trial provided evidence that continued treatment of sertraline (SSRI) attenuates early onset of PPD in women (48). Despite this, further clinical tests are required to define the effectiveness of SSRIs for PPD in compared to other antidepressants.

As expected, it has been demonstrated that serum BDNF levels of women with postpartum affective disorders (depression, manic, and mixed episode) showing suicide risk are lower than those of women showing no risk (49). Interestingly, serum BDNF levels in MDD subjects with longer disease duration is lower in woman, whereas it is no association between serum BDNF and disease duration in men (50). A study investigating a non-synonymous single nucleotide polymorphism (SNP) of valine to methionine at codon 66 (Val66Met) in the BDNF gene revealed a significant association between Met carriers and PPD development when focusing on mothers whose delivery occurred during autumn/winter (51). It has been also reported that serum BDNF levels in male homozygous for Val and female Met carriers are higher than that in male Met carriers and female homozygous of Val (52).

**Figure 1:**
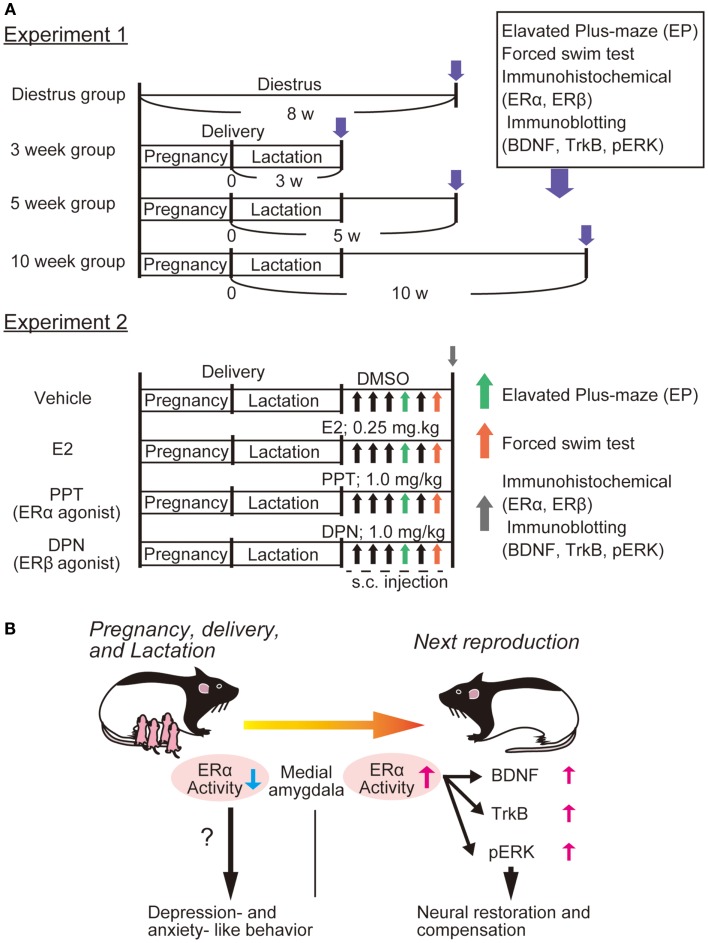
**Possible involvement of ERα function and BDNF-related signaling in PPD**. We recently showed altered expression of ERα and BDNF-related signaling in the rat amygdala [please see Ref. (53)]. **(A)** Experiment 1; four groups were made: control (diestrus nulliparous), and 3-, 5-, or 10-weeks postpartum mother rats were produced. After examining anxiety-like behavior [elevated plus-maze (EPM)] and depression-like behaviors [forced swim test (FST)], immunostaining (for both ERα and ERβ) and immunoblotting (for BDNF, TrkB, and pERK) were performed. Further, to determine which estrogen receptor influences depression-like behaviors most, E_2_, PPT (agonist for ERα), and DPN (for ERβ) (please see Experiment 2) were applied to 3 week postpartum mothers. Behavioral examination with EP and FS tests were carried out at 30 min after the fourth and sixth drug application, respectively. Brain tissues (including amygdala) were also collected for immunochemical analysis following behavioral examination. **(B)** We observed that 3 week postpartum rats exhibited anxiety- and depression-like behaviors. This PPD-like phenotype was associated with reduced ERα (not ERβ) in the medial amygdala [please see Ref. (53)]. It was revealed that both E_2_ and PPT canceled the PPD-like responses, suggesting that the importance of ERα-mediated signaling. We speculated that the function of ERα is important for the ERK pathway (possibly via the upregulation of BDNF/TrkB) and that increased ERα/BDNF-related activity in the amygdala plays a critical role in the neural restoration and compensation to recover from PPD [please see Ref. (53)].

We have observed depressive-like behaviors in primiparous female rats at 3 weeks postpartum compared with nulliparous rats (53). Furthermore, decreased expression of ERα (not ERβ), and increased expression of BDNF and its receptor TrkB in the medial amygdala were observed at 3 weeks postpartum (53). Importantly, administration of 17β estradiol and propyl pyrazole-triol (PPT, selective ERα agonist) in primiparous mothers caused significant upregulation of BDNF, TrkB, and pERK2 in the medial amygdala, in addition to marked improvement of depressive-like behaviors [Ref. (53), see Figure 1]. It is possible that amygdala ERα plays a role in depressive-like behaviors, and that the BDNF/TrkB/ERK system functions to recover from PPD, although further study is required to reveal a relationship between function of ERα and upregulation of BDNF in our model. Interestingly, ovariectomized female rats treated by PPT exhibit anxiogenic behaviors in the elevated plus-maze (EPM) test with increased corticosterone levels, while activation of ERβ by diarylpropionitrile (DPN) reduces anxiety-like behaviors and corticosterone levels (54). Although estradiol has similar binding affinities to ERα and ERβ, subcutaneous injection of estradiol reduces depressive- and anxiety-like behaviors (54). Further, intracerebral delivery of estradiol does not alter anxiety-like behaviors in open field and EPM tests, but increases active stress-coping behavior in the FST (55). Both ERα and ERβ knockout mice are strong tools to investigate the contribution of each estrogen receptor to brain function and molecular regulation including BDNF-related signaling. In the hippocampus of ovariectomized female mice, treatment with estradiol benzoate increased activation of Akt and TrkB, whereas these estradiol effects on Akt and TrkB are abolished in both ERα and ERβ knockout mice (56). Remarkably, upregulation of BDNF mRNA was induced by estradiol treatment in the hippocampus of ERα knockout mice, while ERβ knockout mice showed no alteration in the hippocampal BDNF expression after estradiol exposure, suggesting involvement of ERβ in the regulation of BDNF expression (56).

The lower estradiol and higher progesterone levels in serum were observed in MDD women, which are associated with hypoactivation in the hypothalamus, subgenual anterior cingulate cortex, amygdala, and orbitofrontal cortex (6). A recent cohort study demonstrated a significant inverse association between follicle-stimulating hormone (FSH) and depressive symptoms in women (age 35–47) (57). Estradiol treatment for 12 weeks in postmenopausal women resulted in reduced FSH and luteinizing hormone, and increased dehydroepiandrosterone sulfate (58). Furthermore, the treatment elicited a reduced response to CRH on ACTH-release in the pituitary (58). Estrogen therapy is associated with improvement of PPD and potentiates the antidepressant effect (by sertraline, 50 mg/day) on depressive symptoms in postmenopausal women (59, 60). As these clinical reports suggest that a possible association between estrogen and dysregulation of the HPG- and HPA-axis in women with depression, the interaction among BDNF, estrogen, HPA axis, and HPG-axis is a critical subject when considering treatment for PPD.

## Psychiatric Diseases, Inflammation, and Cytokines

Interestingly, certain signaling pathways downstream of BDNF/TrkB are also activated by cytokines. However, in contrast to purported antidepressant effect of BDNF signaling, cytokine action is largely associated with depressive-like behaviors. The cytokine network is incredibly complex with a plethora of cross-talk between cytokines to regulate activation or inactivation of immune systems (61). Several lines of evidence demonstrate an increase in levels of pro-inflammatory cytokines in the blood and cerebrospinal fluid of patients suffering from psychiatric diseases such as depression and schizophrenia (62–65). Epidemiological studies have also proposed that such inflammation is one of the major risk factors for depression and schizophrenia (66, 67). In a clinical study, cytokine expression caused by lipopolysaccharides (LPS) in monocytes obtained from healthy women was associated with depressive symptoms (68). Experimentally stimulating the inflammatory response in animal models caused behavioral deficits, which are reminiscent of psychiatric diseases.

Administration of LPS or pro-inflammatory cytokines [TNF-α, interleukin-1β (IL-1β), etc.] in rodents acutely triggers a spectrum of behavioral deficits that are known as “sickness-behavior” including diminished feeding, motor activity, and social behavior (9, 69). In addition to sickness-behavior, inflammation induced by LPS or cytokine administration facilitated development of depressive-like behaviors (FST and tail suspension test), and anhedonia (sucrose intake test), with recovery induced by antidepressant treatment (9, 70, 71). Interestingly, indoleamine 2,3-dioxygenase (IDO), a tryptophan-catabolizing enzyme, has been shown to mediate LPS- and complete Freund’s adjuvant-induced depressive-like behavior (72, 73). IL-6 increases hippocampal IDO expression levels with depressive-like behavior in FST (73), suggesting that amino acid metabolism is possibly linked with inflammation-induced comorbid depression.

Cytokine-mediated depressive-like behaviors remain a controversial issue, as these behaviors are considered a rather non-specific phenotype. It has been reported that intracerebroventricular (i.c.v) injection of IL-1β caused both sickness- and depressive-like behaviors along with increases in cortical IL-6 levels, whereas IL-6 application achieved depressive-like behavior (not sickness) but failed to change IL-1β levels (12). This implies that different cytokines are involved in both depression and sickness behaviors, and that IL-6 may contribute to the pathogenesis of depression, though further studies are needed to reveal individual mechanisms pertaining to each respective behavioral pattern.

Pro-inflammatory cytokines also induce activation of intracellular signaling molecules including Janus kinases (JAK), c-jun N-terminal kinase (JNK), p38, ERK, signal transducer and activator of transcription (STAT), and nuclear factor-κB (74, 75). A variety of evidence shows that pro-inflammatory cytokines affect the glucocorticoid receptor (GR) to ultimately influence the HPA axis. An *in vitro* study using mouse fibroblast cells revealed that IL-1 inhibited dexamethasone (an agonist for GR)-induced translocation of cytoplasmic GR to the nucleus, and repressed GR-mediated transcriptional activity (76–78). TNF-α also inhibited gene transcription mediated by GR in HeLa cells through stimulating p38 and JNK pathways (76, 79). Importantly, rats receiving intraperitoneal administration of IL-1 showed increased ACTH and corticosterone (rodent glucocorticoid) in blood due to hypersecretion of CRH in hypothalamic neurons (80). This dysfunction of GR and hypersecretion of CRH after application of pro-inflammatory cytokines might reflect hyperactivation of the HPA axis as observed in depressed patients. In addition to the HPA axis, expression/function of BDNF is also affected by cytokines. For example, a single i.c.v injection of IL-1β upregulated both BDNF and TrkB expression in mouse hippocampus, while daily IL-1β administration (for 8 days) decreased both of these expression levels (81). Moreover, BDNF-stimulated signaling (Akt and ERK, but not PLCγ) was reduced by IL-1β application in cortical neurons (82). Tong et al. found that IL-1β application inhibited facilitation of LTP by BDNF in rat hippocampal slice cultures via suppressing IRS-1/Akt/CREB signaling (83). In their system, an inhibitor for p38 signaling canceled the negative effect of IL-1β on BDNF-mediated signal transduction and LTP, suggesting that p38 signaling plays an essential role in IL-1β function (83). Notably, functional impairments of both GR and BDNF are achieved via activating the identical p38 signaling cascade (78, 79, 83). Further investigation addressing the possible contribution of p38 to the pathogenesis of depressive disorders is needed.

## Putative Involvement of Cytokines in Cancer-Associated Depression

Multiple clinical studies have indicated a high-prevalence rate of depression in patients with cancer (13). Since the discovery that depressive symptoms correlate with shortened survival time in cancer patients (84), the mechanism behind cancer-associated depression has been a critical issue. In addition to the psychological stress of suffering from a life-threatening condition, the physical decompensation caused by cancer itself and/or therapy (including drug treatments) may play a role in comorbid depression. For example, patients undergoing treatment of cancer with IFN-α (a pro-inflammatory cytokine) frequently exhibit depressive symptoms, with cessation of cytokine therapy diminishing symptoms (14). A recent clinical trial demonstrated that IFN-α therapy decreased serum levels of BDNF while increasing depressive symptoms as rated by Montgomery–Asberg depression rating scale in hepatitis C patients (85). The effects of IFN-α administration on behavior or plasma corticosterone levels were relatively mild in rodents compared with that of IL-1β or TNF-α (86). On the other hand, when mice are exposed to psychosocial stressors, IFN-α administration induces a significant increase in plasma cytokine levels (IL-6, -10, and TNF-α), plasma corticosterone concentration and sickness-behavior, suggesting that a synergistic effect of IFN-α with stressors on behavioral and endocrine deficits (87). Remarkably, the direct secretion of cytokines in tumor cells is also involved in cancer-related depressive symptoms as development of depression may be linked to elevated plasma IL-6 and ascites in patient with ovarian cancer (88). Pyter et al. found that rats with mammary tumors caused by *N*-nitroso-*N*-methylurea exhibited depressive- and anxiety-like behaviors (but not sickness), and confirmed increased IL-1β in tumor tissue and elevated levels of cytokines (IL-1β, -6, -10, and TNF-α) in hippocampal regions (89). Lamkin et al. also observed that ovarian carcinoma injected into female mice elicited anhedonic, depressive-like behavior in addition to elevated plasma IL-6, -10, and TNF-α concentrations. These phenomenon caused by cancer transplantation were enhanced after exposure to social isolation stress (90). These animal models are strong tools to clarify the relationship between tumor and depressive-like behaviors, however, detailed molecular and cellular level investigation concerning the impact of tumor-secreted cytokines on CNS neurons is essential for understanding cancer-related comorbid depression.

While estrogen therapy is useful for depressive symptoms in PPD (59, 60), tamoxifen, an antagonist for the ER, is used to treat primary breast cancer with potentially deleterious effect on mood (91). Further, some antidepressants (i.e., fluoxetine, paroxetine, and sertraline) reduce the efficacy of tamoxifen via interaction with cytochrome P450 2D6, an enzyme for tamoxifen metabolism (92, 93), illustrating the challenges associated with treating a patient with MDD and breast cancer.

The role of BDNF as a prognostic marker in breast cancer has been postulated because increased BDNF expression has been found in breast cancer tissue (94). Although low-BDNF expression levels decreased risk for tumor relapse more than high-BDNF levels in breast cancer patients, the risk for early tumor relapse increases from loss of BDNF (95). Because overexpression of BDNF in breast cancer cells resulted in reduction of cell proliferation, Huth et al. proposed a novel role of BDNF as a suppressor of breast cancer (95). To establish a solid treatment method for patients with comorbid disease, the basic mechanism including the interaction between BDNF and estradiol in breast cancer is worth further study.

## BDNF in Obesity and Depression

Metabolic syndrome including hypertension, hyperlipidemia, and central obesity along with diabetes have been identified as risk factors for psychiatric illnesses including depression and schizophrenia (15, 96). A variety of factors including oxidative stress, dysregulation of the HPA axis, imbalance of neurotransmitter systems, inflammation, and neuroprogression including neurodegeneration and reduced neurogenesis, have been proposed to be involved in the pathogenesis of obesity-related mental diseases (15, 96). On the other hand, as BDNF contributes to the regulation of both synaptic plasticity and energy metabolism including feeding behavior (1, 97), the neurotrophin has been recognized as a key target to clarify the relationship between metabolic and psychiatric disease. The latter part of this review covers BDNF and its effect on obesity and psychiatric disease in relation to diet. Importantly, there are many reports and reviews concerning the influence of nutrition, such as omega-3 fatty acids and amino acids, on depression (98, 99). Here, we focus more on the beneficial effects of flavonoids and zinc, as they have the potential to activate the BDNF/TrkB system.

The Val66Met SNP in the BDNF gene has been shown to cause significant dysfunction in dendritic trafficking and activity-dependent secretion of BDNF, ultimately contributing to several neurologic and psychiatric diseases (100). Concerning the relationship between the Val66Met SNP and obesity, an epidemiological study in Korea demonstrated that the body mass index (BMI) in the 66Met allele population is lower compared with that in 66Val (major allele), and that smoking moderated the increase of BMI in both Val/Met and Met/Met genotypes (101). Further, brain imaging and neuropsychological assessments showed reduced cortical volume in obese people with the 66Met allele compared with both healthy subjects and obese people with the 66Val allele (102). In addition, the allele 66Met obesity group displayed significant perseveration on the Wisconsin card sorting test, suggesting that the Met allele in obese populations is a risk factor for dysfunction of the prefrontal cortex (102). Though the detailed mechanisms involved in antipsychotic treatment and development of obesity have not been elucidated, Zhang et al. found that clozapine, an atypical antipsychotic for the treatment of schizophrenia, potentiated the risk of metabolic syndrome (103). A faster increase in glucose plasma levels in the Met/Met genotype compared with that of the Val/Val or Val/Met genotypes in male schizophrenic patients under chronic treatment of clozapine (24 months) was observed, while other metabolic parameters (triglyceride and high-density cholesterol, etc.) showed no significant difference among these genotypes (103). Importantly, this BDNF polymorphism is weakly associated with obesity, though brain-specific conditional BDNF knockout mice exhibit typical symptoms of obesity (hyperglycemia, hyperleptinemia, high cholesterol, and hyperinsulinemia) due to abnormal appetite regulation controlled by both neuropeptide Y (NPY) and pro-opiomelanocortin (POMC) neurons in the hypothalamus (104). Further, increased latency was observed in the light/dark exploration test in BDNF deficient mice, indicating that the deletion of brain BDNF induces both obesity and anxiety (104). It has been reported that anorexigenic neurons in the ventromedial hypothalamus regulate food intake via stimulation of thrombospondin receptor α2δ1, and that the receptor is reduced in conditional BDNF knockout mice (105). Overexpression of the α2δ1 receptor by using the adenovirus gene transfer system partially reversed increased food intake and improved metabolic status such as glucose intolerance in BDNF deficient mice (105). BDNF may have a protective role against the progression of obesity.

Opposite to energy intake, energy expenditure (by exercise, etc.) is considered to improve brain function. In the obese population, decreased expression levels of neurotrophins (NGF and BDNF) and associated receptors (TrkA and TrkB, respectively) elicited by a HFD were observed, through attenuation of neurotrophin downregulation was achieved by training and/or consuming a normal calorie diet (106). A possible mechanism behind exercise-dependent BDNF induction has been demonstrated. Wrann et al. found that exercise upregulated hippocampal BDNF through stimulating peroxisome proliferator-activated receptor gamma coactivator 1-alpha (PGC-1α), which contributes to mitochondrial biogenesis and oxidative metabolism (107). Knockdown of fibronectin type III domain-containing protein 5 (FNDC5), a target gene of PGC-1α, diminished BDNF expression without any influence on other growth factors (CNTF, GDNF, NGF, and IGF-1), suggesting that BDNF plays a role in energy metabolism via the PGC-1α/FNDC5-mediated pathway (107). These reports corroborate the notion that exercise is a beneficial intervention promoting a healthy physical and mental state.

## Altered Synaptic Plasticity in Obesity and Possible Influence of HPA Axis

Recently, impairment of BDNF-related intracellular signaling in metabolic diseases has been demonstrated. In addition to expression of hippocampal BDNF, its receptor TrkB was decreased in leptin deficient mice exhibiting obesity, while levels of NT-3 were intact (16). Hyperglycemia and downregulation of both BDNF and TrkB in leptin deficient mice were recovered by adrenalectomy or low-dose corticosterone replacement (16), suggesting that glycemic status affects the BDNF/TrkB system through the HPA axis. Consistently, Wosiski-Kuhn et al. found that acute decrease of corticosterone by treatment with metyrapone, an inhibitor for corticosterone synthesis, improved deficits in hippocampal expression of BDNF and TrkB in leptin deficient mice (108). They also confirmed reduced hippocampal BDNF and excitatory postsynaptic potential slope after intrahippocampal corticosterone injection (108), implying that chronic GR activation in the hippocampal region impairs synaptic plasticity through repressing the BDNF/TrkB system in metabolic diseases.

Recent research suggests that a HFD can lead to an imbalance of glutamate metabolism in brain tissue (109). A HFD (45 kcal% fat, 35 kcal% carbohydrates, and 20 kcal% protein, for 8 weeks) caused an increase in glutamate uptake efficiency in the hippocampus due to upregulation of glutamate transporters (GLT-1 and GLAST), and impairs hippocampal synaptic plasticity including both basal and NMDA-mediated long-term depression (109). In other research, impaired memory function (novel object recognition) and behavioral flexibility after 8-weeks consumption of a HFD (38 kcal% fat and 38 kcal% refined sugar) were shown (110). Proteomic analysis has also revealed that a HFD affected levels of various proteins in the dorsal and ventral hippocampal regions, including calcium/calmodulin-dependent protein kinase type II (CaMKII) subunit delta and glutamate receptor 3 (GluR3) (110), both involved in hippocampal synaptic plasticity. Importantly, a high-fat sucrose diet (39 kcal% fat and 40 kcal% sucrose) aggravates traumatic brain injury by fluid percussion with reduction of hippocampal BDNF expression (111). Furthermore, reduction of BDNF in the hippocampus and cortex is also involved in HFD (60 kcal% fat)-caused depressive- and anxiety-like behaviors (112, 113). Of note, full-length and truncated types of TrkB (receptor for BDNF) are not downregulated by HFD (113). Although the relationship between obesity and regulators of synaptic function including BDNF remain to be elucidated, current research is underway.

## Action of Flavonoids, Natural Compounds, on Brain Function

Flavonoids are rich in plants and vegetables, and their beneficial functions as cancer-preventing (18) and antioxidant agents (17) have been proposed. For example, quercetin, a typical flavonoid, rescues neuronal cells against oxidative stress induced by H_2_O_2_ exposure through the reduction of reactive oxygen species (114). Nuclear factor E2-related factor 2 (Nrf2)/antioxidant response element (ARE) is critical for the protective action of flavonoids under oxidative insult and is expected to be the potential target for improvement of neurodegeneration (115, 116). Using animal behavioral analysis, studies have shown the antidepressant effect of flavonoids. A significant increase in social interaction time and decreased immobility time in the FST after oral administration of quercetin (20–40 mg/kg) was observed in rats, matching the efficacy of the antidepressant fluoxetine (117). Interestingly, quercetin also attenuates both corticotrophin releasing factor-caused depressive-like behavior and water immersion-restraint stress-induced release of corticosterone (117, 118), strongly suggesting influence on the HPA axis.

**Figure 2:**
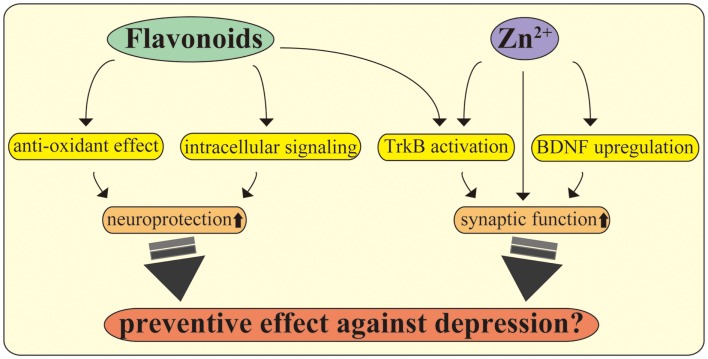
**Flavonoids and zinc exert multiple beneficial effects on CNS neurons**. Flavonoids play multiple roles in the CNS, including promoting antioxidant effects and stimulating intracellular signaling. Further, recent evidence suggests that flavonoids act as TrkB receptor agonists, facilitating neuroprotection and antidepressant effects. The utility of zinc, including modulation of synapses, stimulation of TrkB, maintaining BDNF expression, and exerting antidepressant effects, is also shown. The molecular mechanisms behind the beneficial effects of these nutrients may be a potential target to improve depression in PPD, inflammation, and metabolic diseases.

Current evidence indicates that flavonoids activate intracellular signaling. Using cultured cortical neurons from rats, we recently observed that flavonoids obtained from the plant *Iris tenuifolia* prevent cell death induction by oxidative insult via stimulating phosphorylation of Src homology-2 (SH2) domain-containing phosphatase 2 (Shp2) [Ref. (119), see Figure 2]. Remarkably, Jang et al. found that 7,8-dihydroxyflavone (7,8-DHF), which has been recognized as a new TrkB agonist after screening of various types of flavonoids, exerts potent protective effects equivalent to that of BDNF on hippocampal and cortical neurons (120). In another report, the antioxidant function of 7,8-DHF was also confirmed under oxidative stress caused by H_2_O_2_ exposure (121). Interestingly, studies using conditional TrkB (*Single neuron labeling In Cre-mediated knockout*:*trkB*) or BDNF (*tamoxifen*-*Cre*:*BDNF*) knockout mice have consistently demonstrated flavonoid efficacy (122). English et al. found that administration of 7,8-DHF in fibrin glue promoted axon regeneration in wild-type, but not in TrkB knockout mice. On the other hand, BDNF knockout mice receiving the same treatment showed increased axon length, suggesting that 7,8-DHF has a positive effect on axon profiles via stimulating TrkB even in the absence of BDNF (122). Furthermore, daily injection of 7,8-DHF (intraperitoneal; i.p., 5 mg/kg) induced phosphorylation of TrkB in the hippocampus and improved anhedonia- (sucrose preference test) and depressive-like-behaviors (open-field test) caused by chronic alcohol consumption (19). In limited to the Huntington’s disease mouse model, activation of TrkB leads to significant improvement of motor function, brain atrophy, and survival after treatment with 7,8-DHF (p.o., 5 mg/kg) or by 4′-dimethylamino-7,8-DHF (a derivative of 7,8-DHF., p.o., 1 mg/kg) (123).

## Action of Zinc in Depression

Zinc, one of the essential minerals, has been extensively studied in animal models of depression, demonstrating robust antidepressant properties (124). Szewczyk et al. demonstrated that zinc deficiency increases risk of depression and Alzheimer’s disease, while supplementation of zinc is an effective treatment of these disorders (125). Consistently, zinc deficiencies were observed in 41.0% of psychogeriatric patients including depressive disorders, compared to 14.4% of the control group demonstrating this deficiency (126).

Intracellular Zn^2+^ concentration is maintained through activity of zinc transporters consisting of Slc30a (ZnT1-10; exporting cytosol Zn^2+^ to organelles or outside of the cells) and Slc39 (ZIP1-14; passing Zn^2+^ into cytosol) families (127, 128), each affecting synaptic function of glutamatergic neurons (129–131). In hippocampal mossy fibers, the zinc transporter-3 (ZnT-3) plays a role in recycling zinc to stimulate ERK via inhibiting tyrosine phosphatase activity, resulting in positive regulation of memory function (129). In dendritic spines of hippocampal neurons, zinc transporter-1 (ZnT-1) has also been found to contribute to synaptic zinc homeostasis (130). Zrt/Irt-like protein 12 (ZIP12) stimulates CREB and promotes neurite outgrowth (131). Interestingly, G-protein coupled receptor 39 (GPR39; recognized as a zinc receptor) is expressed in neuronal cells (132). Synaptic zinc evokes synthesis of endocannabinoid 2-arachidonoylglycerol (2-AG) through stimulating GPR39, leading to decreased presynaptic neurotransmitter release (133). Furthermore, zinc also functions as an agonist for TrkB and potentiates LTP of CA3 synapses in hippocampal mossy fibers (134). Concerning cell survival, it has been reported that increased apoptotic cell death in hippocampal regions (including CA1, CA3, and dentate gyrus) and decreased phosphorylation of hippocampal TrkB and ERK were observed in lactating zinc deficient (0.85 ppm) mice (135). Importantly, a zinc deficient diet (0.2 and 1 ppm) for 3 and 6 weeks caused depressive and anxiety-like behaviors in addition to anhedonia (136–138), while these depressive-like behaviors were reversed after chronic treatment with typical antidepressants (such as fluoxetine, imipramine, and reboxetine) (136, 138). In addition, such zinc deficiency in the diet induced downregulation of GPR39, BDNF, and its receptor TrkB in both hippocampal and cortical regions when compared with adequate zinc (33.5 ppm) (138). All these studies suggest that the possibility of zinc homeostasis is associated with mood status and BDNF-related neuronal function.

## Conclusion

**Figure 3:**
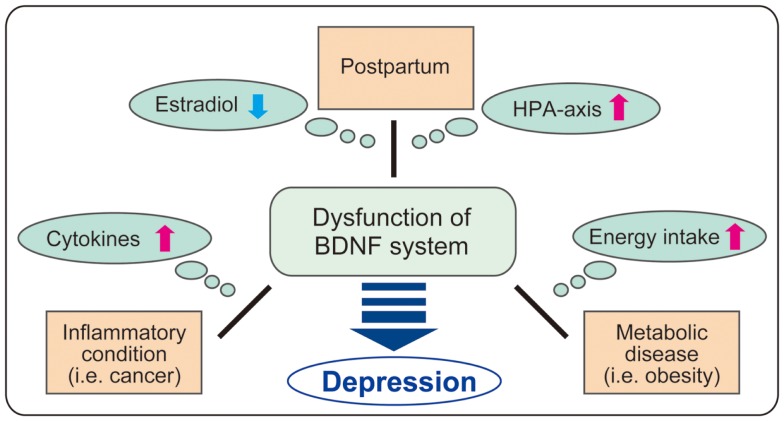
**Possible role of BDNF in comorbid depression**. It is possible that BDNF system is impaired in comorbid depression in postpartum, inflammatory conditions including cancers, and metabolic disease through the change of various factors (estradiol, HPA axis, cytokines, and energy intake). As BDNF regulates a variety of brain functions, further investigation on BDNF dysfunction in each condition such as postpartum, cancers, and obesity may contribute to clarify the complex mechanism in comorbid depression.

It has been suggested that both BDNF and glucocorticoid regulation are potential targets of therapy for major depression. In this paper, the molecular dynamics including downregulation of BDNF in brain tissues and altered activity of the HPA axis in PPD, inflammatory conditions, and metabolic disease are discussed (see Figure 3). Investigation concerning the intricate relationship between major factors (estrogen, glucocorticoids, cytokines, BDNF, etc.) and their specific role in the development of depression is critical. Basic analysis using gene-deficient/overexpression mice combined with exposure to other factors may be attractive. For example, further examination of cytokine administration using brain-specific GR- or BDNF-deficient mice may reveal detailed linkage among glucocorticoids, BDNF, and inflammation. As shown in the latter part of the paper, current studies demonstrate the beneficial effects of flavonoids and zinc, both acting as TrkB agonists. While several reports demonstrate the protective effects of flavonoids and zinc against metabolic disease (139, 140), effects of these nutrients on depression in PPD or inflammation-related diseases remains to be elucidated. To truly understand comorbid depression due to inflammation and metabolic disease, more detailed investigation of peripheral metabolism and brain function is needed.

## Conflict of Interest Statement

The authors declare that the research was conducted in the absence of any commercial or financial relationships that could be construed as a potential conflict of interest.
